# Preliminary outcomes of a paediatric highly active antiretroviral therapy cohort from KwaZulu-Natal, South Africa

**DOI:** 10.1186/1471-2431-7-13

**Published:** 2007-03-17

**Authors:** Anand Reddi, Sarah C Leeper, Anneke C Grobler, Rosemary Geddes, K Holly France, Gillian L Dorse, Willem J Vlok, Mbali Mntambo, Monty Thomas, Kristy Nixon, Helga L Holst, Quarraisha Abdool Karim, Nigel C Rollins, Hoosen M Coovadia, Janet Giddy

**Affiliations:** 1 Sinikithemba HIV/AIDS Clinic, McCord Hospital, Durban, South Africa; 2 Children's Rights Centre, Durban, South Africa; 3 CAPRISA, Nelson R. Mandela School of Medicine, University of KwaZulu-Natal, Durban, South Africa; 4 Department of Community Health, Nelson R. Mandela School of Medicine, University of KwaZulu-Natal, Durban, South Africa; 5 Department of Paediatrics and Child Health, Nelson R. Mandela School of Medicine, University of KwaZulu-Natal, Durban, South Africa; 6 Doris Duke Medical Research Institute, Nelson R. Mandela School of Medicine, University of KwaZulu-Natal, Durban, South Africa

## Abstract

**Background:**

Few studies address the use of paediatric highly active antiretroviral therapy (HAART) in Africa.

**Methods:**

We performed a retrospective cohort study to investigate preliminary outcomes of all children eligible for HAART at Sinikithemba HIV/AIDS clinic in KwaZulu-Natal, South Africa. Immunologic, virologic, clinical, mortality, primary caregiver, and psychosocial variables were collected and analyzed.

**Results:**

From August 31, 2003 until October 31, 2005, 151 children initiated HAART. The median age at HAART initiation was 5.7 years (range 0.3–15.4). Median follow-up time of the cohort after HAART initiation was 8 months (IQR 3.5–13.5). The median change in CD4% from baseline (p < 0.001) was 10.2 (IQR 5.0–13.8) at 6 months (n = 90), and 16.2 (IQR 9.6–20.3) at 12 months (n = 59). Viral loads (VLs) were available for 100 children at 6 months of which 84% had HIV-1 RNA levels ≤ 50 copies/mL. At 12 months, 80.3% (n = 61) had undetectable VLs. Sixty-five out of 88 children (73.8%) reported a significant increase (p < 0.001) in weight after the first month. Eighty-nine percent of the cohort (n = 132) reported ≤ 2 missed doses during any given treatment month (> 95%adherence). Seventeen patients (11.3%) had a regimen change; two (1.3%) were due to antiretroviral toxicity. The Kaplan-Meier one year survival estimate was 90.9% (95%confidence interval (CI) 84.8–94.6). Thirteen children died during follow-up (8.6%), one changed service provider, and no children were lost to follow-up. All 13 deaths occurred in children with advanced HIV disease within 5 months of treatment initiation. In multivariate analysis of baseline variables against mortality using Cox proportional-hazards model, chronic gastroenteritis was associated with death [hazard ratio (HR), 12.34; 95%CI, 1.27–119.71) and an HIV-positive primary caregiver was found to be protective against mortality [HR, 0.12; 95%CI, 0.02–0.88). Age, orphanhood, baseline CD4%, and hemoglobin were not predicators of mortality in our cohort. Fifty-two percent of the cohort had at least one HIV-positive primary caregiver, and 38.4% had at least one primary caregiver also on HAART at Sinikithemba clinic.

**Conclusion:**

This report suggests that paediatric HAART can be effective despite the challenges of a resource-limited setting.

## Background

In 2005, an estimated 2.3 million children were living with human immunodeficiency virus/acquired immunodeficiency syndrome (HIV/AIDS) worldwide; 700,000 became newly infected, and 570,000 died [1]. Trials in the United States and Western Europe have demonstrated that highly active antiretroviral therapy (HAART) is effective in suppressing HIV viral replication and reversing immunodeficiency in children [2,3]. The result has been a reduction in paediatric hospital admissions and a decrease in morbidity and mortality due to HIV/AIDS [4-7].

South Africa is home to an estimated 230,000 children infected with HIV/AIDS [8]. Statistics suggest that 50,000 of these children qualify for HAART by World Health Organization (WHO) staging, while only 7,000 have been initiated by the national antiretroviral roll-out program [8]. Despite this large paediatric HIV population, research on care and treatment in this setting is limited. Numerous studies have confirmed the clinical efficacy and feasibility of HAART in adult HIV patients in Africa [9-13] but to date very limited experience address the use of paediatric HAART in Africa. Msellati and colleagues in Côte d'Ivoire, Mbaye et. al in Senegal, Eley et. al., Cowburn et al, and van Kooten Niekerk et al., in South Africa, and the AIDS Working Group of Medecins Sans Frontieres report that good paediatric outcomes can be achieved in Africa [14-19].

Resource-limited settings pose unique challenges to the implementation, effectiveness, and sustainability of HAART programs. Limited availability of paediatric formulations of antiretroviral drugs, weak healthcare infrastructure and the widespread conviction that paediatric specialists are needed to initiate and monitor HAART due to its perceived complexity serve as barriers to comprehensive implementation [20,21]. Threats to effectiveness and sustainability in the paediatric population include morbidity and mortality of primary caregivers due to rising HIV prevalence rates, and although the expectation of poor adherence in this setting leading to widespread "antiretroviral anarchy" has not been met, treatment failure and exhaustion of available regimens are ongoing risks [22].

The present study describes a paediatric HAART cohort in KwaZulu-Natal, South Africa. We report preliminary outcomes for 151 children initiated during a 25 month period.

## Methods

### Setting

KwaZulu-Natal (KZN), the most populous province in South Africa, has the highest HIV-1 adult prevalence rate with the antenatal seroprevalence at 40% [23]. Tuberculosis (TB) incidence in this setting is 782.2 per 100,000, and two thirds of HIV patients are co-infected with TB[24]. Fifty percent of the paediatric hospital admissions and 40% of the under-five mortalities in KZN are HIV related [25,26]. Sixty nine percent of children in this province live in poverty, and more than half the children live in households with inadequate water supplies and sanitation [27].

McCord Hospital is a state subsidized urban hospital in Durban, KZN. In 2004, McCord Hospital's Sinikithemba ("We give hope" in IsiZulu) clinic received funding from the United States' President's Emergency Plan for AIDS Relief (PEPFAR) fund for HAART scale-up. This funding subsidizes treatment for all patients. The paediatric co-payment is ZAR 60/month (~ US$10) and treatment is free for children whose parents are also receiving HAART at Sinikithemba. Patient co-pay includes all HAART and prophylaxis medication, clinic visits, and laboratory testing allowing all patients in the program to receive 6 monthly CD4 and viral load testing.

Sinikithemba clinic has designed and implemented a *family centered *model of care. All adults patients accessing HAART are routinely interviewed about their children's health, and those at risk are referred for testing and care. Reciprocally, some children receiving HAART at Sinikithemba have precipitated the diagnosis and subsequent initiation of treatment for their infected primary caregivers. Referred family members are prioritized for HAART enrollment, and families are given clinic appointments for the same day when possible.

### Patient selection, preparation, and follow-up

Clinical and psychosocial selection criteria are identical to the South African Department of Health guidelines for care and treatment of HIV-infected children [28]. To qualify for HAART at Sinikithemba paediatric patients must be symptomatic (WHO clinical stage 3 or 4) or have a CD4% of < 20% in a child < 18 months of age or a CD4% of < 15% in a child > 18 months of age[29]. In addition there must be at least one caregiver who is able to supervise the child's medication and disclosure to another adult living in the same household. The one requirement unique to Sinikithemba is the self-reported ability of the primary caregiver to fund treatment monthly. Patients over the age of 16 years were referred to Sinikithemba's adult clinic.

In a process modeled after the South African national program, patient preparation includes three education sessions for caregivers on HIV, HAART and the importance of adherence, as well as a thorough clinical assessment of the child. Baseline laboratory tests include CD4%, full blood count, liver function tests, urea and electrolytes, and a chest x-ray and/or ultrasound/sputum (when necessary to exclude TB). If there are no clinical problems preventing the initiation of HAART, it is prescribed on the day the caregiver finishes the third training session. Antiretroviral therapy is optimally delayed by 2 months for children who present with TB at baseline [30]. Children return for follow-up visits with doctors 2 weeks post HAART initiation and monthly thereafter.

### Treatment

The South African National Paediatric Antiretroviral Guidelines are used[30], though only brand-name medications are purchased due to PEPFAR's trademark drug regulations. First line, second line, and TB-concurrent HAART regimens are summarized in results. (Some children had regimens other than the South African national plan.) All children are prescribed cotrimoxazole prophylaxis until their CD4% is consistently > 20% for > 6 months. Ritonavir replaces Kaletra™ for the duration of concurrent TB treatment in children < 3 years.

### Clinical measurements

Weight and height are measured at every clinic visit. Children prescribed Kaletra™ also receive a baseline and 6-monthly fasting cholesterol, glucose, and triglycerides assay [30,31]. No baseline viral load (VL) is performed due to cost constraints. CD4% and VL are performed every 6 months in all children. CD4 lymphocyte counts were performed by flow-cytometry using the FACSCalibur (Becton Dickinson, San Jose, CA, USA) and VL were determined with the Amplicor HIV-1 Monitor PCR test v1.5 (Roche Diagnostics Systems, Branchburg, NJ, USA), with a lower limit of detection of 50 copies of HIV RNA/mL.

### Study design and definitions

This was a retrospective cohort study. The study population consisted of all paediatric patients who were eligible for HAART at Sinikithemba from August 31, 2003 until October 31, 2005. Data were collected from clinical charts and from the hospital's electronic patient database system. Deaths were reported by family members to the clinic by use of verbal autopsy [32].

Clinical diagnoses of chronic gastroenteritis and TB were made in accordance with WHO and the South African Department of Health paediatric clinical guidelines [28-30]. *Pneumocystis carinii pneumonia *(PCP) was confirmed by chest x-ray and/or (when available) sputum induction for stains to detect *Pneumocystis *carinii. Lactic acidosis in our clinic is defined as a lactate of > 5 mmol/l and an arterial blood bicarbonate level of < 20 mmol/L (or a total venous C02 < 20 mmol/l) with other causes such as septiceaemia and dehydration excluded [33].

Treatment failure that resulted in a change in regimen was defined as a VL > 5000 copies of HIV RNA/ml, for greater than 6 months, resulting in an immunological decline. Genotypic resistance testing was not available to our paediatric cohort due to cost.

A primary caregiver was recorded as a person who has consistently assumed responsibility for the housing, health, or safety of the child [34]. Typically, these were the individuals who completed pre-HAART training, administered medication daily and attended clinic appointments. There was no requirement that the caregiver be the patient's only caregiver or that he/she live with the child. Primary caregivers were designated during a psychosocial intake interview, and changes were noted by clinicians during patient visits. One to three primary caregivers were recorded for each child.

Adherence was assessed by monthly patient self-report of the primary caregiver and child [35,36]. A treatment interruption due to poor adherence was defined as three or more consecutive missed daily doses in a treatment month.

Lost to follow-up was defined as no communication between the patient and the clinic staff regarding whereabouts resulting in missing at least 3 monthly follow-up appointments and/or failure to collect medication[37].

### Statistical analysis

Data was analyzed using SAS version 9.1 (SAS Institute Inc., Cary, NC). Patients lost to follow-up or a change in service provider was censored at time of event; while all other patients were censored at time of last clinic visit through October 31, 2005. Date of death was taken as endpoint for those patients who died. Changes in median CD4% were compared by Student's t-test. Weight-for-age Z-scores (WAZ) and Height-for-age Z-scores (HAZ) over time were compared using the Kruskall-Wallis test. WAZ and HAZ scores were calculated by EpiInfo version 3.3.2 based on the Centers for Disease Control (CDC) standardized weight and height curves. The Cox proportional-hazards model was used to assess the relationship between baseline variables and mortality. Variables statistically significant (p < 0.05) in univariate analysis were subsequently tested in multivariate analysis. Kaplan-Meier survival methods were used to estimate the probability of survival from the time of initiation of antiretroviral therapy to death.

### Ethics

The research ethics committee of McCord Hospital and the scientific review committee of CAPRISA, University of KwaZulu-Natal approved this study.

### Sources of Funding

United States' President's Emergency Plan for AIDS Relief (PEPFAR) fund administered by the Elizabeth Glaser Paediatric AIDS Foundation (EGPAF). PEPFAR funding was only used for care and treatment of paediatric patients. PEPFAR and EGPAF did not contribute intellectually towards this publication.

## Results

### Study cohort

From August 31, 2003 until October 31, 2005, 151 children initiated HAART at Sinikithemba. Thirteen children died during follow-up (8.6%), one changed service provider, and no children were lost to follow-up. Median follow-up time of the cohort after HAART initiation was 8 months (IQR 3.5–13.5).

### Baseline characteristics (presented in Table 1)

The majority of the cohort had advanced HIV disease at initiation of treatment as indicated by their WHO clinical stage, CD4% (n = 146, 5 missing values), and baseline anthropometry.

Fifty children (33.1%) had TB at baseline: 35 pulmonary, 13 extrapulmonary and two concurrent pulmonary-extrapulmonary.

At commencement of HAART, 119 children (78.8%) were antiretroviral naïve, 19 (12.6%) had received HAART previously at another site, and 13 (8.6%) had a failed prevention of mother-to-child transmission (PMTCT) intervention.

Initial HAART regimens of the cohort are presented in Table 2.

### Primary Caregivers

**Figure 1:**
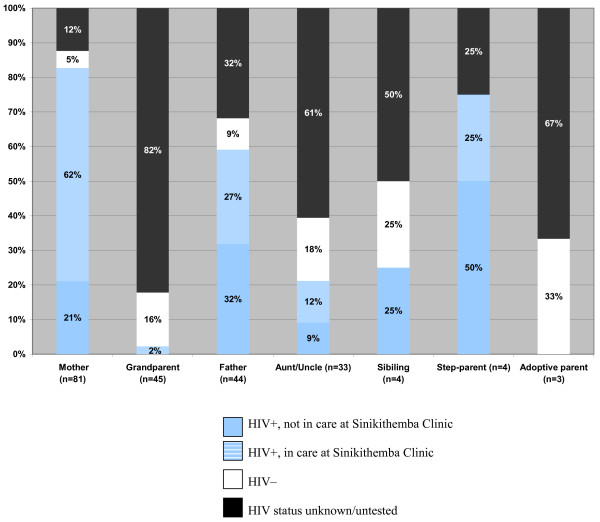
**HIV status and HAART in the primary caregivers of the Sinikithemba paediatric cohor**: The Sinikithemba cohort was cared for by 214 familial and non-familial primary caregivers. Of these 214 primary caregivers; 105 (49.1%) had tested positive for HIV of whom 68 (64.8%) were also receiving HAART at Sinikithemba, 86 (40.2%) were unaware of their status, and 23 (10.7%) had tested HIV negative. Ten children were in institutional care (orphanage), no primary caregiver identified.

The 151 children were cared for by 214 familial and non-familial primary caregivers as presented in Figure 1. The distribution of HIV-positive caregivers was not limited to a few households as 52.3% of children had at least one HIV-positive caregiver, and 38.4% of children had at least one primary caregiver also in care at Sinikithemba.

### Treatment responses

**Figure 2:**
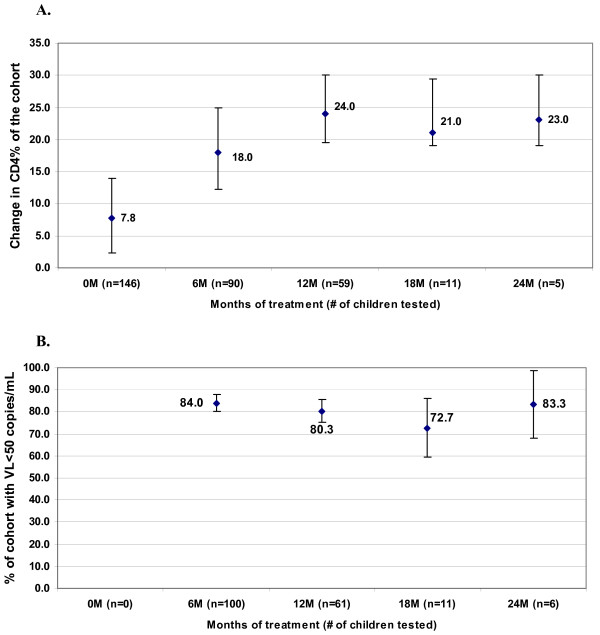
**CD4% and Viral Loads changes of the Sinikithemba paediatric cohort in response to HAART**: A. Median increase in CD4% from baseline. The vertical bars in subfigure A indicate IQR. B. Percentage of cohort achieving undetectable viral loads. The vertical bars in subfigure B indicate 95% CI.

Immunologic and virologic responses to HAART are presented in Figure 2. Children that initiated HAART in the 6 months prior data censure did not have a 6-monthly CD4% and VL result available. All children reaching a 6 month or 12 month endpoint had CD4% and VL. The cohort's median change in CD4% from baseline was 10.2 (IQR 5.0–13.8) at 6 months (p < 0.001), and 16.2 (IQR 9.6–20.3) at 12 months (p < 0.001). At the time of publication VLs were available for 100 children at 6 months, of which 84% (95%CI 80.3–87.7) were undetectable. At 12 months 61 children had available VLs, and 80.3% (95%CI 75.2–85.4) of these were undetectable. The cohort's median changes from baseline to last available WAZ and HAZ scores were 1.0 and 0.4, respectively, after an average of 8.9 months. The change was statistically significant for WAZ (Kruskall-Wallis test p < 0.0001) but not for the HAZ score (Kruskall-Wallis test p = 0.2880). Sixty-five out of 88 children (73.8%) reported a significant increase (p < 0.001) in WAZ score after the first month.

### Mortality

In univariate analysis of baseline variables against mortality, significant hazard ratios were associated with WHO clinical stage 4, weight-for-age < fifth percentile, and baseline presence of: TB, chronic gastroenteritis, pneumonia, and PCP. Loss of one or two parents was not significantly associated with mortality. In multivariate analysis, chronic gastroenteritis was significantly associated with death and the presence of an HIV-positive primary caregiver was found to be *protective*. These data are presented in Table 3.

**Figure 3:**
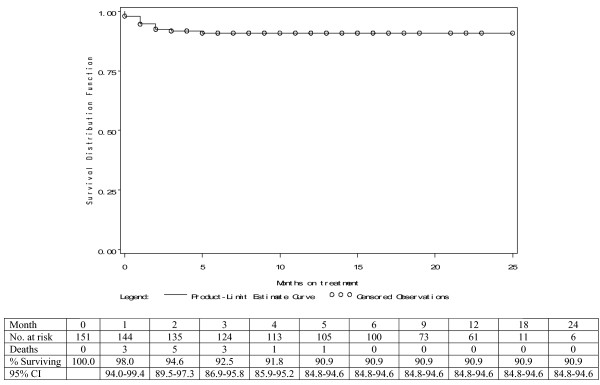
Kaplan-Meier mortality survival curve of the cohort.

The Kaplan Meier (presented in Figure 3) mortality survival estimate at 12 months was 90.9% (95%CI 84.8–94.6). Paediatric mortalities are summarized in Table 4. All 13 deaths occurred within the first 5 months of HAART initiation. No deaths were attributed to metabolic disorders or other drug-related adverse effects. Autopsy data were not available, but the most commonly reported causes of death were chronic gastroenteritis (n = 6) and TB (n = 4). Other causes of death included: sepsis syndrome (n = 1), suspected PCP (n = 1), and a respiratory tract infection (n = 1).

### Regimen durability

One hundred and thirty-four patients (88.7%) are still on their first Sinikithemba HAART regimen. Fifteen (9.9%) have progressed to a second line, one (0.7%) to a third line, and one (0.7%) to a fourth line. Of the 17 (11.3%) that had a regimen change, only two were due to antiretroviral toxicity. One child discontinued zidovudine because of anemia, and one child stopped stavudine because of lactic acidosis. Seven regimen changes due to treatment failure were recorded. Initiation or completion of TB treatment resulted in six children changing regimens. Two children switched from Kaletra™ to Efavirenz with increasing age (> 3 years).

### Adherence

Ninety patients (59.6%) reported no missed doses for the duration of their treatment. Forty-two (27.8%) children reported "some missed doses" during at least one month of treatment, not exceeding 2 missed doses per month (> 95% adherence). Sixteen (10.6%) reported one or more treatment interruptions. Adherence data was missing for 3 children. Common reasons reported for missed doses were: financial trouble that prevented caregivers from collecting medication on time, vomiting of medication without re-dosing, incorrect dosing by a caregiver, missed clinic appointments and pharmacy collections, confusion between multiple caregivers, and child refusal or self-discontinuation.

### Disclosure

At the time that the data was censored, 12 (7.9%) children had been made aware of their own HIV+ status: 0% of children < 3 years of age (n = 0), 2.4% of children 3–5 years (n = 1), 7.5% of children 6–8 years (n = 3), 25% of children 9–11 years (n = 3) and 83% of children 12–15 years (n = 5). Children who had not been disclosed to were given partial, inaccurate, or no information by caregivers.

## Discussion

### Limitations

The Sinikithemba self-pay requirement may have resulted in a selection bias towards patients with greater financial resources. However, their decision to seek semi-private subsidized care may instead reflect limited access to HAART in the public sector, where province-wide paediatric enrollment is stunted due to long waiting lists, staff shortages, laboratory backups, etc. Fifty-six percent of our cohort is living well below the national poverty line, as indicated by their eligibility for a government welfare grant which requires a combined household income of less than ZAR 800/month (~ US$134). More research is necessary to understand the financial constraints of this population, but limited validity of this data due to financial inequity cannot be assumed.

CD4 and VL laboratory results were available for all children who reached a 6 or 12 month endpoint, however no values were recorded for patients initiated in the 6 months prior to data censure. The follow-up time of the cohort was insufficient to confirm the long-term efficacy of HAART in this setting, although good short-term outcomes are indicated. To date this paediatric cohort is the largest in Africa to be documented in the literature; however, there were relatively few deaths to be included within the univariate and multivariate analyses.

### Analysis

We have documented the initial experiences of 151 paediatric HIV patients initiating HAART in KZN, South Africa. Our data suggests that a family-centered paediatric HAART program in a resource-limited setting can be both clinically effective and sustainable as demonstrated by therapeutic responses, survival, patient retention, and regimen durability.

The Sinikithemba paediatric cohort's immunologic and anthropometric responses to HAART were comparable to the other paediatric cohorts in Africa as well as the United States and Europe [14-16,38]. Eighty-six out of 90 (95.6%) children at 6 months and 57 out of 59 (96.6%) children at 12 months had an increase in CD4% from baseline. In developing-world settings where CD4 monitoring is not available due to cost, weight has been used as a prognostic indicator of clinical effectiveness of HAART [39,40]. By this measure, the Sinikithemba cohort demonstrated an immediate and significant response to therapy: 65 out of 88 children (73.8%) reported a significant increase in WAZ score after the first month following HAART. The significant change in the cohort's WAZ scores but not HAZ scores is to be expected given the abbreviated follow-up time of our study [14,41,42].

The Sinikithemba cohort exhibited high degrees of virologic suppression: 84.0% at 6 months and 80.3% at 12 months. In Côte d'Ivoire, 49.3% of children (n = 73) achieved virologic suppression(< 250 copies/mL) after 12 months [14]. In South Africa, after 12 months of HAART 69.7% of children (n = 264) had a viral load < 400 copies/ml [43]. In the United States in a multi-site paediatric cohort of 263 children, Ruthstein et al reported a rate of 34% virologic suppression (< 400 copies/mL) at 12 months[44]. Both the Côte d'Ivoire (mean age: 7.2 yrs, age range 0.7–15.2 yrs) and the cohort reported by Ruthstein et. al (mean age: 8.5 yrs, age range 0–17 yrs) had similar age ranges compared to the Sinikithemba cohort. There are differences between the Sinikithemba cohort and other paediatric studies (such as different populations, viral subtypes, HAART regimens, etc.), which render conclusions drawn from direct comparisons to be guarded. It may also be possible that the Sinikithemba cohort has higher rates of adherence which may contribute to more effective virologic suppression [45,46]. More research is necessary to investigate the sources of this discrepancy.

The Sinikithemba mortality rate of 8.6% compares favorably with other paediatric cohorts in the developing world (ranges 7.0%-20.0%) [14-16]. Although we have shown that the baseline presence of many WHO clinical stage 4 symptoms have a higher hazard ratio for mortality; age, orphanhood, baseline CD4%, and hemoglobin were not predicators of mortality in our cohort as they were in other published African cohorts [9-11,47,48].

No children were lost to follow-up, despite much of the cohort having to contend with long travel distances, multiple caregivers, and self-funding responsibilities. Adequate follow-up data from other paediatric cohorts is not available; however, this rate contrasts sharply with data from African adults cohorts, which have a weighted mean of 79.8% patient retention at 12 months[12]. There are several plausible reasons for this. The immediate improvement in most of the cohort (nearly three-quarters reporting significant weight gain in the first month) may have convinced caregivers of the effectiveness of HAART and the need for ongoing care. Finally, treating caregivers and children at the same site may create stronger ties to families resulting in better retention.

Harries et al. summarize the clinical arguments against the sustainability of HAART by advising HAART programs in resource-limited areas that they would be "prudent to decide what drugs might be used for salvage antiretroviral therapy *when *(author's emphasis) drug resistance, side-effects, or both become a problem."[22] However, only two children at Sinikithemba (1.3%) experienced an adverse event that required a regimen change, a rate similar to paediatric results from Côte d'Ivoire and substantially lower than the percentages recorded for African adult cohorts (range 14.3%-80.2%) [12,14]. We have not conducted structured adherence analysis, but patient self-report data, high rates of viral suppression, and low incidence of treatment failure indicate that adherence levels were not a threat to regimen durability or an obstacle to good clinical outcomes in this cohort [45].

Other paediatric HAART studies in Africa have analyzed the rates of orphanhood within their cohorts, but to our knowledge none have recorded the HIV-status of the primary caregivers or analyzed the outcomes associated with HIV-positive caregivers [34]. Our data shows that although more than half of the Sinikithemba cohort is cared for by at least one HIV-positive caregiver, these caregivers showed a *protective *effect against mortality when compared with caregivers who were untested or HIV negative. We hypothesize that HIV-positive primary caregivers on HAART at the same site may be able to provide more informed treatment support for their children resulting in better outcomes. Those HIV positive caregivers not yet on HAART may still be more knowledgeable about symptom management and disease progression, more experienced with health care systems and/or more likely to access available health services. Further research is necessary to investigate these possibilities, but the necessity of supporting all caregiver/dependent relationships is clear.

## Conclusion

Many settings in Africa, with social and economic constraints placed on children, demand thoughtful and sensitive management of HIV-positive children on HAART. Despite this, we have shown that a cohort with advanced HIV disease in this setting responds favorably to treatment.

All 13 deaths occurred in children with advanced HIV disease within 5 months of treatment initiation. This suggests that a greater effort should be made to identify and treat HIV-positive children at an earlier stage of their infection, and that children with advanced disease should be closely monitored and supported at the onset of HAART. Based on these data we suggest that these children, especially those who present with chronic gastroenteritis which may interfere with uptake/absorption of the medications, be referred to paediatric inpatient wards or a local palliative (step-down) care center for HAART initiation. Treatment counselors can conduct home visits to monitor and support caregivers, especially those with little treatment experience.

A third of our cohort presented with TB at baseline and four deaths were TB related. With such a high prevalence in this setting, more research on paediatric HIV/TB co-infection is necessary, especially with regard to immune reconstitution syndrome, multi-drug resistance, and best-practice guidelines for concurrent and/or sequential HAART/TB treatment in order to minimize drug toxicity and optimize outcomes [9,24].

The high rate of HIV positive caregivers indicates that it is imperative to transform the paediatric treatment model from child-centered to family-centered in order to protect the integrity of caregiving structures and prevent the negative paediatric outcomes associated with the declining health or death of a primary caregiver [48]. Recruiting untested caregivers for testing and extending treatment opportunities to the caregivers who test positive is a necessary step towards protecting the future health and well-being of the paediatric patient.

## Competing interests

The author(s) declare that they have no competing interests.

## Authors' contributions

All authors read and approved the final manuscript. AR, SCL, and JG conceived the project. AR and SCL collected and analyzed the data, and co-drafted the manuscript which was commented on by all the authors. AG was the statistician for this project. RG, QAK, and NR throughout the study provided critical discussions on the manuscript. KHF, GLD, WJV, MM were the physicians that treated the children. MT developed the adherence counseling program for the clinic. KLN was the manager of the monitoring and evaluation program in the clinic. HLH and HMC wrote the grant to obtain funding for HAART scale-up. HMC and JG were senior contributors for this project and contributed equally.

## Pre-publication history

The pre-publication history for this paper can be accessed here:



## Figures and Tables

**Table 1:** Baseline characteristics of the Sinikithemba paediatric cohort

**Demography**	**[n (%)]**
**Children**	151
**Female**	77 (51.0%)
**Age**	
< 3 years	39 (25.8%)
3–5 years	43 (28.5%)
6–8 years	43 (28.5%)
≥ 9 years	26 (17.2%)
Median years [range]	5.7 [.3–15.4]
**Parent status**	**[n (%)]**
Two parents alive	67 (44.4%)
Only father living	19 (12.6%)
Only mother living	27 (17.9%)
Neither parent living	38 (25.1%)
**WHO clinical stage **[n (%)]	**[n (%)]**
Stage 1	26 (17.2%)
Stage 2	19 (12.6%)
Stage 3	72 (47.7%)
Stage 4	34 (22.5%)
**Anthropometry**	**Median (IQR)**
Weight-for-age Z-score	-1.9 (-3.6 to -0.9)
Height-for-age Z-score	-2.2 (-3.0 to -1.1)
**CD4 lymphocyte %**	**[n (%)]**
< 5%	52 (35.6%)
5%-9%	36 (24.7%)
10%-14%	28 (19.2%)
15%-24%	26 (17.8%)
> 25%	4 (2.7%)
Median CD4% (IQR)	7.4 (2.1–13.7)
**Laboratory values**	**Median (IQR)**
Albumin (g/L)	30.0 (26.0–35.0)
ALT (IU/L)	18.0 (14.0–28.3)
Hemoglobin (g/dl)	10.1 (8.9–11.0)
**Tuberculosis**	**[n (%)]**
Present	50 (33.1%)
**Other opportunistic infections**	**[n (%)]**
Chronic gastroenteritis	52 (34.4%)
Pneumonia	13 (8.6%)
Oesophageal candidiasis	13 (8.6%)
PCP	11 (7.3%)
Cryptococcal meningitis	1 (0.7%)
CMV retinitis	0
Toxoplosmosis	0

**Table 2:** Initial Antiretroviral Regimens

Age	Regimen	NRTIs	NNRTI/PI			
			EFV	NVP	Kaletra™	RTV

< 3 years (n = 39)	1^st ^line	d4T/3TC	7	1	23	0
	2^nd ^line	AZT/ddI	0	0	0	0
	TB	d4T/3TC	0	0	0	5
≥ 3 years (n = 112)	1^st ^line	d4T/3TC	61	0	1	0
	2^nd ^line	AZT/ddI	1	0	1	0
	TB	d4T/3TC	37	0	1	2

ARV, antiretroviral therapy

d4T, stavudine; 3TC, lamivdine; AZT, zidovudine;

ddI, didanosine; EFV, efavirenz; RTV, Ritonavir

**Table 3:** Mortality: Cox proportional hazards models between baseline characteristics and death

		**Univariate analysis**		**Multivariate analysis**	
**Baseline Characteristic**	**No. of Deaths**	**Hazard ratio 95% CI**	***p***	**Hazard ratio 95% CI**	***p***

**Gender**					
Male	6	1.00		1.00	-
Female	7	1.14 (0.38–3.40)	0.810	2.78 (0.66–11.86)	0.163
**Age**					
< 3 yrs	5	1.00		1.00	-
3–5 yrs	2	0.35 (0.07–1.81)	0.210	1.37 (0.21–9.04)	0.747
6–8 yrs	3	0.52 (0.12–2.16)	0.365	0.25 (0.02–2.93)	0.268
≥ 9 yrs	3	0.92 (0.22–3.85)	0.908	2.18 (0.44–10.85)	0.342
**WHO clinical stage**					
Stage 1–3	7	1.00		1.00	-
Stage 4	6	3.01 (1.01–8.95)	0.048*	1.82 (0.41–8.03)	0.430
**Weight **(**2 missing values)					
≥ 5 Percentile	1	1.00		1.00	-
< 5 Percentile	10	8.60 (1.10–67.15)	0.040*	4.96 (0.45–55.31)	0.193
**CD4% **(**1 missing value)					
≥ 5%	5	1.00		-	-
< 5%	7	2.65 (0.84–8.34)	0.097	-	-
**Tuberculosis**					
Absent	5	1.00		1.00	-
Present	8	3.39 (1.11–10.37)	0.032*	1.08 (0.25–4.59)	0.919
**Pulmonary**					
Absent	9	1.00		**-**	**-**
Present	4	1.53 (0.47–5.01)	0.480	**-**	**-**
**Extrapulmonary**					
Absent	10	1.00		**-**	**-**
Present	3	3.06 (0.84–11.20)	0.091	**-**	**-**
**Chronic gastroenteritis**					
Absent	3	1.00		1.00	**-**
Present	10	6.69 (1.84–24.30)	0.004*	12.34 (1.27–119.71)	0.030*
**Pneumonia**					
Absent	9	1.00		1.00	**-**
Present	4	5.23 (1.61–16.98)	0.006*	4.03 (0.79–20.54)	0.094
**PCP**					
Absent	10	1.00		1.00	-
Present	3	3.81(1.05–13.86)	0.042*	5.35 (0.84–33.86)	0.075
**Oesophageal candidiasis**					
Absent	12	1.00		-	-
Present	1	0.92 (0.12–7.04)	0.932	-	-
**Hemoglobin**					
> 10 g/dl	5	1.00		-	-
≤ 10 g/dl	8	1.87 (0.61–5.73)	0.272	-	-
**Primary caregiver HIV Status**					
HIV negative or unknown	10	1.00	**-**	1.00	-
HIV positive	3	0.26 (0.07–0.95)	0.041*	0.12 (0.02–0.88)	0.037*
**Primary caregiver receiving HAART at Sinikithemba**					
Primary caregiver not in care	12	1.00	-	-	-
Primary caregiver in care	1	0.13 (0.02–1.01)	0.051	-	-
**Parent status**					
Two parent alive	6	1.00	-	-	-
One parent alive	4	0.92 (0.26–3.26)	0.896	-	-
Neither parent living	3	0.82 (0.21–3.28)	0.778	-	-

**Table 4:** Baseline biological characteristics of children who died

**Pt#**	**Sex**	**Age (Yrs)**	**WHO Stage**	**WAZ**	**CD4%**	**ARV Regimen**	**ARV time to death (weeks)**	**ALB(g/L)**	**ALT(IU/L)**	**HGB(g/dl)**	**Suspected cause of death**
1	M	0.3	4	-	37.6%	d4T/3TC/Kal	09	25.0	14.0	9.9	Chronic gastroenteritis
2	F	7.5	4	-9.52	7.9%	AZT/3TC/EFV	20	18.0	33.0	10.7	TB Miliary
3	F	9.8	4	-4.34	0.8%	d4T/3TC/EFV	14	30.0	12.0	7.9	TB Adenopathy
4	M	3.0	4	-4.81	-	AZT/3TC/Kal	10	-	-	9.1	TB Lymph
5	F	7.1	3	-5.94	8.0%	d4T/3TC/EFV	06	32.0	24.0	11.7	Chronic gastroenteritis
6	F	0.8	3	-7.50	6.0%	d4T/3TC/Kal	01	26.0	116.0	7.0	Chronic gastroenteritis
7	F	1.5	4	-5.42	27.0%	d4T/3TC/RTV	05	28.0	53.0	10.2	TB Abdomen
8	F	13.4	3	-9.51	2.1%	d4T/3TC/EFV	02	-	-	10.1	Chronic gastroenteritis
9	M	0.5	4	-5.24	4.0%	d4T/3TC/Kal	05	25.0	45.0	11.7	Chronic gastroenteritis
10	M	6.3	3	-	1.1%	AZT/3TC/EFV	05	39.0	16.0	9.3	Chronic gastroenteritis
11	M	12.2	3	-6.95	1.0%	d4T/3TC/EFV	06	-	-	5.6	Suspected PCP
12	F	1.1	3	-0.87	1.0%	d4T/3TC/Kal	01	19.0	8.0	4.1	Sepsis syndrome
13	M	5.2	3	-6.71	0.5%	d4T/3TC/EFV	08	28.0	8.3	7.0	Respiratory tract infection

d4T, stavudine; 3TC, lamivdine; AZT, zidovudine; ddI, didanosine; EFV, efavirenz; RTV, Ritonavir; Kal, Kaletra™
